# Integration of Fiber-Optic Sensor Arrays into a Multi-Modal Tactile Sensor Processing System for Robotic End-Effectors

**DOI:** 10.3390/s140406854

**Published:** 2014-04-16

**Authors:** Peter Kampmann, Frank Kirchner

**Affiliations:** 1 DFKI GmbH, Robotics Innovation Center (RIC), Robert-Hooke-Str. 1, Bremen D-28359, Germany; E-Mail: frank.kirchner@dfki.de; 2 Robotics Group, Department of Mathematics and Computer Science, University of Bremen, Robert-Hooke-Str. 1, Bremen D-28359, Germany

**Keywords:** multi-modal tactile sensing, tactile sensor processing, local pre-processing

## Abstract

With the increasing complexity of robotic missions and the development towards long-term autonomous systems, the need for multi-modal sensing of the environment increases. Until now, the use of tactile sensor systems has been mostly based on sensing one modality of forces in the robotic end-effector. The use of a multi-modal tactile sensory system is motivated, which combines static and dynamic force sensor arrays together with an absolute force measurement system. This publication is focused on the development of a compact sensor interface for a fiber-optic sensor array, as optic measurement principles tend to have a bulky interface. Mechanical, electrical and software approaches are combined to realize an integrated structure that provides decentralized data pre-processing of the tactile measurements. Local behaviors are implemented using this setup to show the effectiveness of this approach.

## 1. Introduction

Over the last few years, there has been a growing effort to increase the autonomy of robotic systems to get them into unstructured environments out of factory buildings. One requirement for this striving for long- term autonomy is to sense the environment with massive density and with different modalities. Until now, the sense of touch has been heavily under-represented in robotic systems.

Cutkosky *et al.* [1] describe in their work how a combination of different sensing modalities for touch leads to a multi-modal tactile sensing system that ideally supports manipulation, exploration and response to objects in the contact area of a robot. Several conclusions can be derived from the model described in their work: A single modality for measuring contact is not sufficient to measure all variations of forces. Furthermore, the amount of sensors that are needed poses a challenge in integrating these into robotic systems, because many sensing elements have to be placed directly at the contact area.

There exists a variety of developments on multi-modal sensing systems in the state of the art. However, none of these are sufficient for the system described in [1]. The developed tactile sensing system described in this paper has been designed to meet these requirements.

This paper presents the selection of the measurement principle for each modality and discusses the integration of the system into a three-fingered robotic manipulator. A measurement concept, whose application is rather unusual in robotic systems, has been chosen for the force sensing array. The mechanical-, electronic- and software-wise integration of the selected fiber-optic sensor array in the finger structure of our gripper will be discussed in detail.

In the following chapters, an overview on the state of the art for multi-modal sensing systems and optical measurement principles in robotic applications is given. This section is followed by the presentation of the developed tactile sensing system and the motivation for the selection of measurement principles for each modality. The main focus in this paper is on the tactile sensor array, where a fiber-optical sensing system has been chosen. The measurement principle, the integration and the algorithms to analyze the sensor data are presented. This paper concludes with several experiments to characterize the performance of the measurement method together with examples that show the power of sensor data pre-processing to handle the increasing complexity of robotic systems

### 1.1. State of the Art

Many different approaches tackle the task of tactile sensing for robotic systems. There exists a variety of different sensing principles, and more and more types of tactile sensor arrays have been developed. A recent overview of the general development of tactile sensors can be found in [2,3].

The integration of multi-modal tactile sensing systems is especially relevant for the approach discussed in this paper. A multi-modal tactile sensing system does not rely on a single sensing principle to gather information like contact forces, geometry and slippage. Like the biological archetype, there exist measurement principles that are especially good at sensing one modality of forces. One example for this is the use of force sensing resistor arrays for the detection of contact information and absolute forces. The measurement of absolute forces requires the precise calibration of all sensor elements in the array, which, in most cases, comes with a high effort, as this procedure has to be repeated frequently. A force-torque sensor would be a more suitable sensor for measuring the absolute forces. For sensing the force distribution, on the other hand, the force sensing resistor array is a good solution, while a single force-torque sensor would not be sufficient for realizing this task.

The following subsection will focus on the state of the art for multi-modal tactile sensors. Afterwards, the state of the art in fiber optic tactile sensing in robotic end-effectors will be discussed, as the sensing principle described in this paper has not been used for this purpose before.

#### 1.1.1. Multi-Modal Tactile Sensing in Robotic End-Effectors

A modular multi-modal tactile sensing module, called Hex-o-skin, is presented in [4]. The developed hexagonal modules are equipped with four phototransistors, a three-axis accelerometer and seven temperature sensors. With the described setup, the sensor modules are able to sense pre-touch, light touch, vibration and temperature. The signal processing is done directly at the sensing area; communication within the sensing system is realized using *Universal Asynchronous Receiver Transmitter* (UART) as the transport layer.

The work in [5] presents a finger-shaped sensor module that is able to sense contact forces, micro-vibrations and thermal fluxes, known under the name, BioTac [6].

Sensing these modalities is realized by various sensors in the finger structure. The inner part of the sensor is filled with a fluid that is electrically conductive. As soon as the sensor comes into contact with an object, the increase of pressure is transmitted to the pressure sensor at the base of the finger. Several electrodes in the structure measure the increase of conductivity at the contact area. A thermistor in the fingertip is used for pre-heating the conductive fluid in order to measure the temperature transport that is influenced by the object in contact with the sensor.

In [7], a multi-modal force sensing system is presented that is designed for measuring both dynamic forces and contact forces. The setup consists of a sensing element with piezoelectric sensing properties made of polyvinylidene-fluoride (PVDF) together with a force sensing resistor array. The whole setup is designed for usage in the finger structure of the SKKU [8] hand.

Besides the presented tactile sensing systems, other multi-modal tactile sensor systems have been developed to prove new fabrication methods or sensing principles ([9,10]). It will be interesting to see the integration of these sensors on robotic end-effectors.

#### 1.1.2. Fiber-Optic Force Sensing in Robotics

In [11], a fiber optic tactile sensor is proposed for the integration into the fingertips of robotic grippers.

The measurement principle operates in the following manner. Light is directed into an acrylic plate serving as an optical wave-guide. The rubber sheet on top of the plate is equipped with conical-shaped feelers that collapse when there is contact with an object. At these points, light is reflected out of the acrylic plate. The bright spots at the contact regions are monitored by a CCD camera below the shown setup.

An array of fiber-Bragg-grating sensors for tactile sensing is presented in [12]. The designs that have been tested use either diaphragms or bridges as transducers.

Both approaches haven been realized, building a field of three by three sensing elements. The system has been tested on robotic end-effectors. An on-board processing system has not been realized.

The work in [13] presents a fiber optic sensing principle that is combined with local pre-processing within the gripper structure.

Each sensing element consists of a photo-reflector that is emitting light into the foam structure above and detecting the amount of light that is reflected by the foam.

## 2. Developed Tactile Sensing System

The tactile system was designed with the goal of gathering as much information as possible about the grasped object only by touch. Similar to the ability of humans, who are able to recognize objects in known environments simply by touch in pure darkness, the robotic system should be able to operate under limited visibility, which is necessary for occluded operation areas or in deep-sea, where, until now, hazy water conditions have forced operators to stop their manipulation tasks.

**Figure 1.**
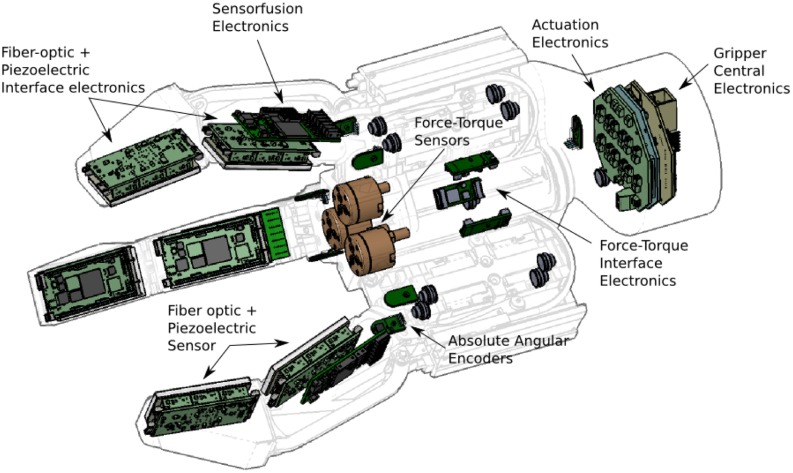
Proposed tactile sensing processing system.

Figure 1 shows the realized tactile sensing system. Force sensing arrays have been placed on each limb of the gripper in order to sense the force distribution while in contact with an object. This information is combined with a dynamic force sensor array that gives information about possible slippage or the texture of the grasped object. The dynamic force sensor has been integrated on the same contact area as the force sensing array. As force arrays tend to be rather difficult to calibrate in terms of measuring absolute incoming forces, an absolute force sensing module is integrated into the finger base.

By choosing dedicated measurement principles for each of the described modalities, we expect higher quality and confidence in the sensor feedback as compared to using a single measurement principle, where other force modalities have to be derived by calculation (e.g., absolute forces through calibration and integration of the sensor element feedback).

## 3. Sensor Selection

As discussed in the previous chapter, suitable sensors for measuring absolute forces, as well as sensors for measuring static and dynamic force distributions have to be selected.

For measuring absolute forces, a six degrees-of-freedom force-torque sensor using strain gauges as transducers has been chosen. The sensor is placed in the finger base of each finger. Using this configuration, the so-called intrinsic tactile sensing [14] cannot be applied, as the sensor element has to be placed in the finger tip in order to avoid ambiguities in the calculation of the incoming forces. However, by merging the force-torque sensor values with the contact information of the sensor array at the corresponding finger, this drawback can be compensated for.

The force-torque sensors are interfaced by a custom pcbcontaining a mixed-signal processor from Cypress. The strain gauge signals are acquired via an external ultra low-noise ADC from Analog Devices (AD7194).

**Figure 2.**
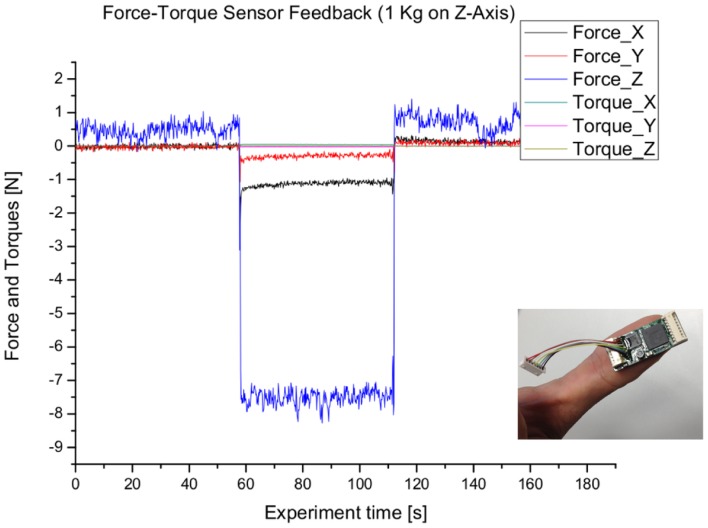
Output of the force-torque sensor using custom interface electronics (lower right corner).

Although the original calibration of the force-torque sensor is lost due to shortening the connection cable and changing the interface connector, this system gives reasonable feedback while applying forces to it. Figure 2 shows the feedback of the force-torque sensor while being loaded with 1 kg in the z-axis direction.

Piezoelectric sensors are used in various applications as sensing devices for dynamic impacts. In order to sense the direction of dynamic forces, an array made of piezoelectric ceramics has been developed. Like for the force-torque sensor array, the interface electronics is realized using a mixed-signal processor from Cypress. These highly integrated chips include programmable analog components, like programmable gain amplifiers and analog multiplexers, as well as configurable analog to digital converters.

**Figure 3.**
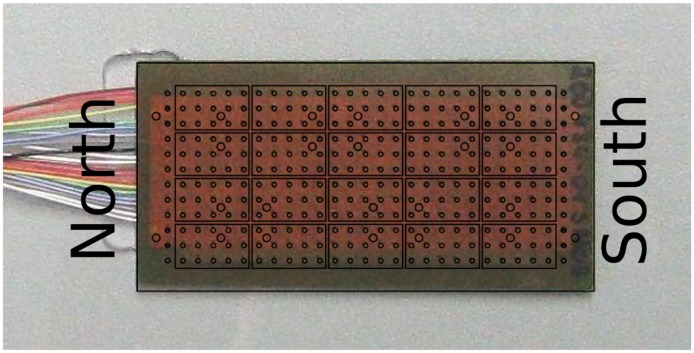
Structure of the custom designed piezoelectric sensor element.

Figure 3 shows the realized piezoelectric sensor array. It has a dimension of six by three centimeters. On this area, there exist 20 sensing elements set up by five sensor elements in a row and four per column.

**Figure 4.**
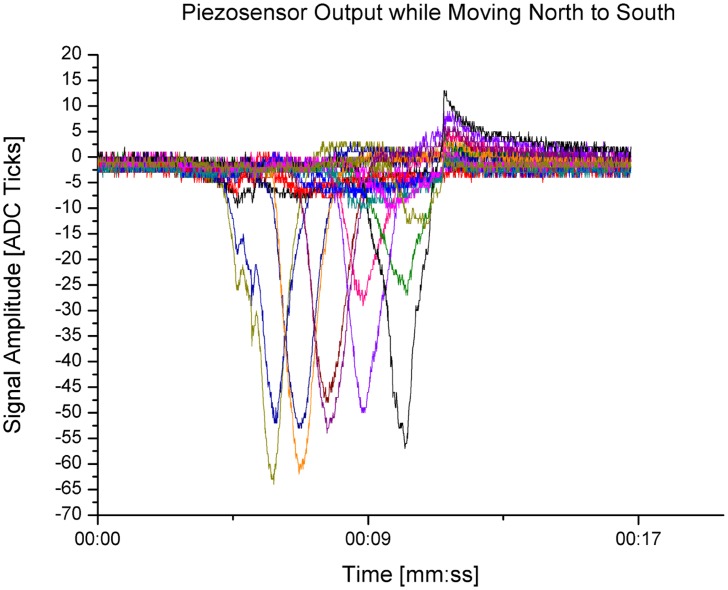
Piezoelectric sensor output while moving the object north to south.

An example of the sensor output of the piezo-sensor array is shown in Figure 4. A cylindrical-shaped object has been moved from north to south in the middle of the sensor plane. In the diagram, this movement can be seen by the consecutive rising and falling sensor element feedback.

As discussed in Section 1.1.2., the use of optical measurement principles in tactile sensor arrays requires either space for complex electronics or space for the lens system of a camera. Especially for tactile sensor arrays that are considered for use in robotic structures, the integration setups tend to be a challenge concerning the available area in end-effector modules.

However, there exists a fiber optic sensing principle for which a space-saving integration approach has been designed. Originally, this sensor type had been developed for the Canadian Space Agency as a collision detection sensor for the Canadarm of the International Space Station, as well as for airbag deployment techniques [15]. Further details on the working principle and the processing interface will be discussed in the following sections and can be found in [16].

Table 1 summarizes the discussed sensor selections.

The original motivation for selecting the presented sensors has been an application scenario in deep-sea environments. Therefore, the sensing principles had to be chosen in a way that the ambient pressure induced by the water column does not influence the measurement principle. This is given for all selected sensors. Like the piezoelectric material, the strain-gauges integrated in force-torque sensors do not exert any signal while in ambient pressure. For the fiber-optic measurement principle described in more detail below, we have proven that this is working under a high ambient pressure of up to 600 bar [17].

Besides the functionality on land and under water, all the selected measurement principles are also likely to work in space environments. This makes this setup a universal tactile sensing solution for all kinds of environments.

## 4. Fiber Optic Measurement Principle

**Figure 5.**
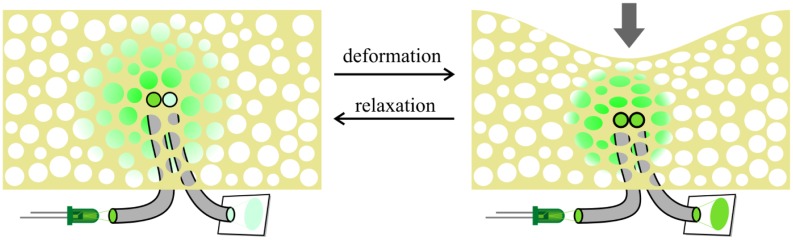
The working principle of the fiber optic sensor.

The sensing principle is realized using foam and polymer-optic fibers. Figure 5 shows the measurement setup.

A pair of optical fibers form a sensor element. One of these fibers is emitting light into a foam cell structure that is placed on top of the fibers. The emitted light is scattered in the cell structure of the foam and is received by the second fiber. As soon as the foam gets compressed due to contact with an object, the way the light is scattered in the foam changes. When increasing the pressure, the area where the emitted light is reflected is reduced, and more light is collected by the sensing fiber.

The received signal is a variation in light intensity, which now needs to be transferred into an electrical signal. Although converting the force signal twice (at first, into light intensity and then into a digital signal) seems rather complicated, this procedure has some advantages compared to a direct signal conversion. The polymer-optic fibers, which are used to transport the light signal, have a low attenuation, which allows the transmission of the signal for some distance. In case there is no space for a direct conversion, this property can be used to transmit the signal to a place in the robotic system where there is more space. Using this setup, the contact sensing area is completely free of electronics. This is helpful for certain applications, like in medical environments, and allows the sensor to get in contact with water without having the risk of a short-circuit. Another advantage of using an optical system within the tactile system is that this approach is not prone to electrical noise and, thus, does not influence or get influenced by other sensors or actuators.

The next section of this chapter will focus on the development of small interface electronics for the described measurement principle.

### 4.1. Interfaces

The signal conversion electronics for fiber optic sensors is, in most cases, bulky; either due to the complex setup of the signal conversion or due to the distance to the object that is necessary for lens-based systems. In the end-effectors of robotic systems, there is not much room for integrating such interfaces. The end-effector size is adapted to the objects that should be manipulated. The space within the end-effector is filled with actuation modules, electronics and sensing modules for proprioceptive and exteroceptive information. Adding the interface electronics for tactile sensors into that space implies an integration challenge.

Until now, the described fiber optic sensor system has been interfaced by using single photo-transistors or arrays of them. Their analog voltage output has to be converted to digitally processable signals. According to [13], this setup is difficult to install on a robot and requires large space.

A more compact approach is to connect the polymer optic fibers to a highly compact array of photo-transistors, like as can be found in camera chips [18]. While other optic measuring principles require the use of lenses in order to monitor the sensing area, it is possible to attach the polymer optic fibers directly to the camera chip surface and, thus, reduce the size of the setup. The size of the sensing area, which can be monitored by this approach, is dependent on the resolution of the tactile array, the diameter of the polymer optic fibers and the size of the camera sensor chip.

**Figure 6.**
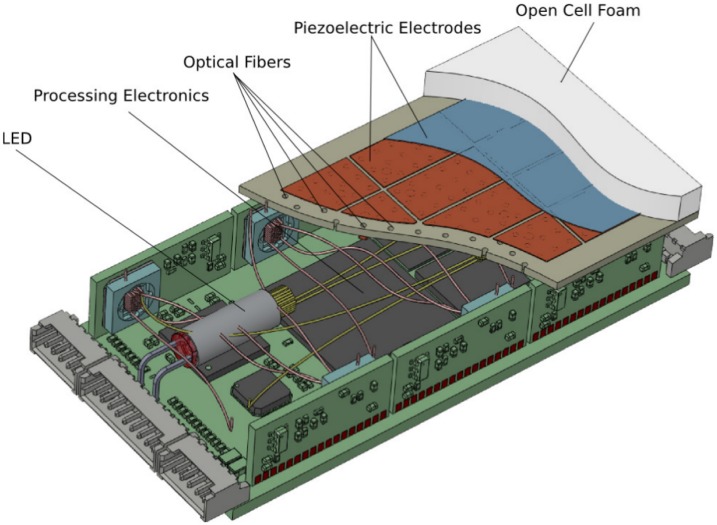
A drawing of the multi-modal tactile sensing module for a robotic gripper.

Figure 6 shows the realized sensor system. The sensing area is six by three centimeters in size. Altogether, there are up to 324 fiber optic sensing elements, which are interfaced by six camera modules, each having a chip size of 5.08 cm. The camera chips have a resolution of 640 × 480 pixels and are capturing the scenery at 30 frames per second.

The fiber optic measurement principle is combined with the piezoelectric sensor array mentioned in Section 3 and enables the measurement of static and dynamic forces on the same area. This is realized by placing the piezoelectric ceramic material on the base-plate that keeps the optical fibers perpendicular to the open cell foam structure. One electrode of the piezo-ceramics has been laser-cut into rectangular parts (marked red in the drawing in Figure 6) to realize a piezoelectric sensor array. After manufacturing, holes are drilled into the piezoelectric material for the polymer-optic fibers of the optical sensor array.

### 4.2. Parametrization

Several parameters do exist to tailor the measurement principle to the specific needs of the application. The sensor readout frequency can be adjusted by the frame rate of the capturing camera. Consumer camera chips often do not run faster than 30 fps, but there exist camera chips that capture image frames with a frequency of more than 1,000 Hz. An example of such a chip is the Hamamatsu S9132, which is capable of running with up to 3,200 frames per second, having a sensing area of 256 × 256 pixels. The amount of sensor elements than can be interfaced with a single camera depends on the active chip size of the camera sensor. Currently, a camera chip of a 1/6-inch active sensing size is used. This chip can capture 60 sensor elements when using polymer optic fibers with a diameter of 250 *μ*m.

Changing the sensitivity of the sensor can be achieved in several ways. A solution that can be used at the runtime of the sensor is changing the parameters of the camera gain and exposure. Another way is to change the brightness of the light source for the emitting fibers. The type of foam that is used also plays an important role in the sensitivity of the sensory system. Depending on the compression load deflection, which gives information about the compressibility of the foam, the sensor varies in its sensitivity.

A similar behavior can be observed when changing the hardness of the protective cover of the sensor.

## 5. Algorithms

**Figure 7.**
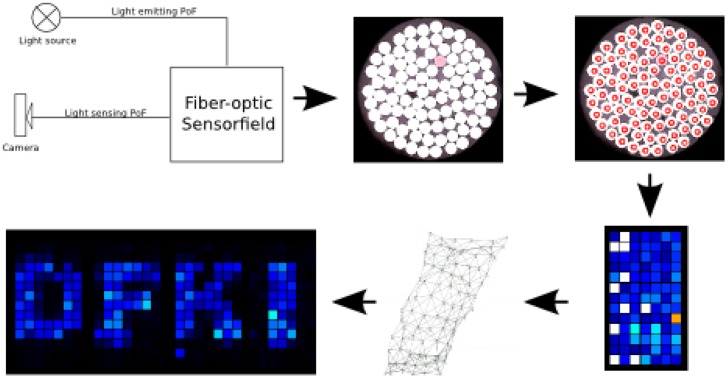
Processing flow for the fiber optic sensor.

Having captured the force signal that is acquired by the fiber optic sensor is only the first step in a series of steps to receive a tactile sensor image of the contact area. The complete processing flow is depicted in Figure 7.

The processing starts in the top left corner, where the signal acquisition is shown schematically. Starting from that, the camera image capturing the attached polymer-optic fibers has to be processed. A simple algorithm that can be easily implemented on embedded hardware, like FPGAs, has been developed to extract the brightness information for every sensor element (see Section 5.1).

In order to keep the mechanical integration as simple as possible, the optical fibers are attached to the camera chip in complete disorder. This means that at this point of the processing flow, the heat map representation of the tactile image (shown in the lower right corner in Figure 7) is also disordered. Several steps have to be done to restore the correct neighborhood relations of single sensor elements. At first, each sensor element is checked for its signal span. If this is below 90% of the average signal span, these sensor modules are considered as not working and are marked accordingly in the software representation.

**Figure 8.**
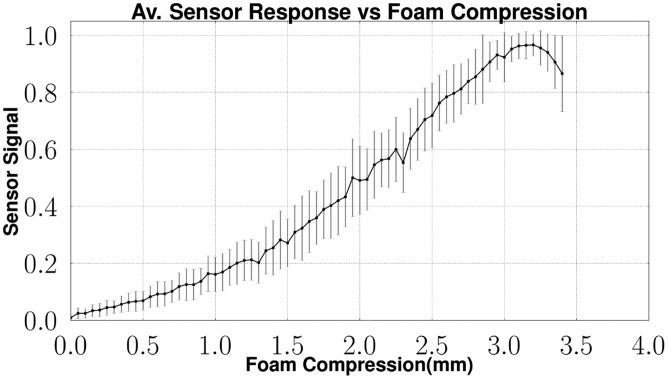
Sensor feedback depending on the applied compression of the foam.

Depending on the cell structure of the foam at the sensing point, variations of the sensor signal span can occur. This is why the sensor elements have to be equilibrated. This step is done to ensure a unique scaling of the signal span of each sensor. The equilibration process is done by measuring the no-load signal offset of each sensor element and the full excitation signal. As the relation for the signal span is linear (compare Figure 8), a simple scaling factor is calculated.

Having identified damaged sensor elements and the signal span for each sensor element, the neighborhood relation can be detected. The algorithm is described in Section 5.3.

Special attention has been paid to the possibility of realizing all algorithms on an embedded device, such that they are implementable on a local processing unit. Furthermore, all calibration routines do work without a special external tool. This allows for ease in the field calibration or recalibration and, eventually, a self-calibration of the robot if the necessary dexterity is available.

The steps that are necessary to receive a tactile image of the contact area, starting at the acquired camera image, are described in the following sections.

### 5.1. Optical Fiber Detection

The optical fiber detection describes the steps that are needed to extract the brightness information of each sensor element from the camera image.

**Figure 9.**
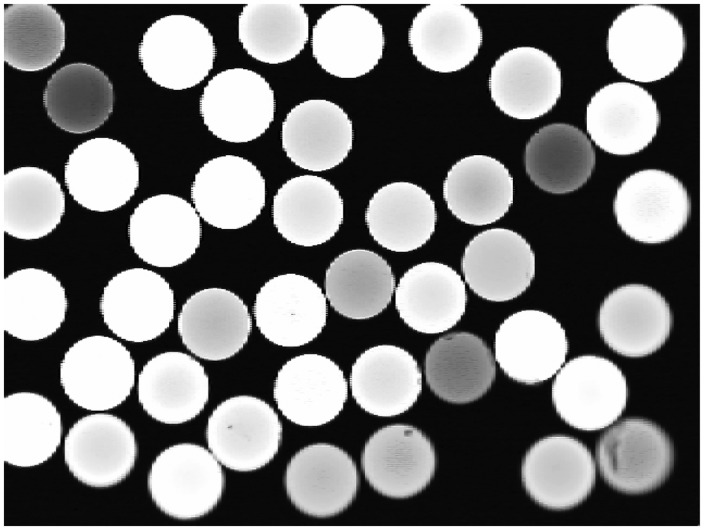
Camera output of the sensor system.

An example camera image is shown in Figure 9. The white circles in the image denote the fiber endings of the sensing polymer optic fibers that are attached to the foam of the sensor. The varying brightness of these endings correspond to a different intensity of the scattered light.

As each optical fiber is covered by more than one pixel on the CMOS sensor, there exists a high amount of redundant information in a captured image. The information should be reduced to the brightness information that can be encoded in one variable for each of the optical fibers.

In order to realize this, the optical fibers need to be extracted from the camera image. A straightforward approach would be using traditional computer vision algorithms that are designed for detecting circles in images, like the Hough transform for circles. This approach is not ideally suited for the use on embedded systems, that have limited resources, as the whole camera image has to be stored during the execution of the algorithm. Because the potential input images, as shown in Figure 9, are of good quality regarding the contrast and noise, a more dedicated algorithm has been developed that is tailored to the characteristics of the images [19].

**Figure 10.**
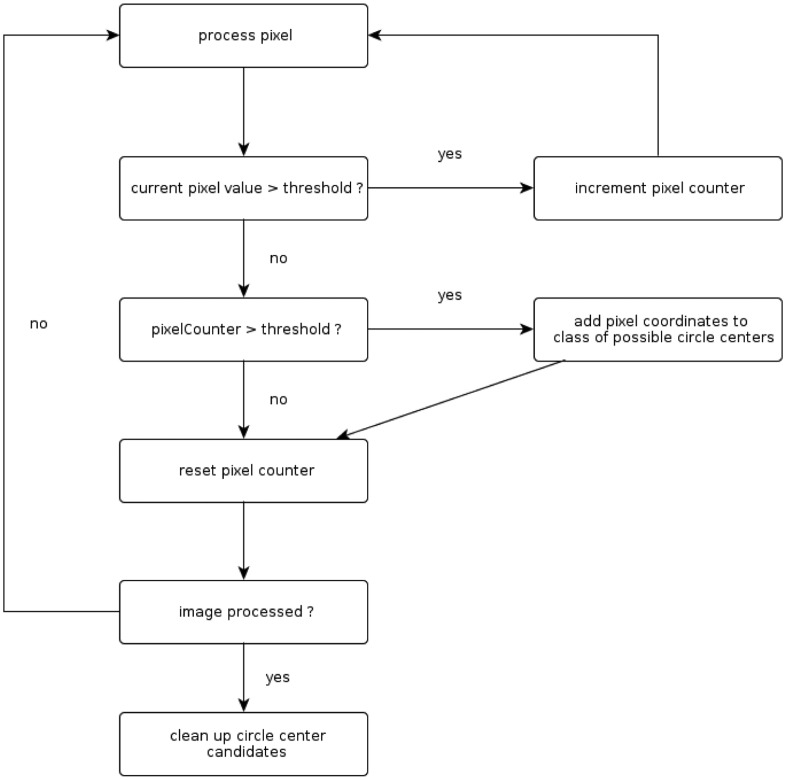
Working principle of the algorithm to detect optical fibers.

The work flow of the algorithm is visualized in Figure 10. The described approach exploits the fact that the image information is given out pixel by pixel through the camera module. Unlike algorithms, like the circular Hough, this approach can process the picture while it is captured. The basic idea behind the algorithm is to look for the longest line of consecutive pixels that have a brightness value higher than a certain threshold. The longest of those lines in one circle is the line of the circle center. When dividing the line length by two, the coordinates of the circle center can be obtained. This approach is very easy to implement on embedded hardware, like FPGAs (8% slices used on a Spartan 6 LX25), as it is not memory consumptive—a complete picture does not need to be stored—as well as mathematically easy. Furthermore, it is even faster than a circular Hough approach with the given image properties [19]. When having calculated the coordinates for the center of each optical fiber, the brightness information at this coordinate is considered as the value of this sensor element. By exploiting the redundant information for each fiber through combining the brightness values of several pixel values, the robustness of the sensor data acquisition is increased and the influence of noise from the CMOS sensors is reduced.

### 5.2. Calibration

Several material properties of the foam have an influence on the signal span and signal offset of a sensor element. One parameter is the alignment of the optical fibers with respect to each other. The fibers are glued together and placed into the holes of the carrier plate. Due to imprecision during the manufacturing process, it cannot be ensured that the sensing and emitting fiber in every pair are aligned completely in parallel at the sensing area. A variation of the orientation of the two fibers leads to different reaction characteristics of a sensor element.

**Figure 11.**
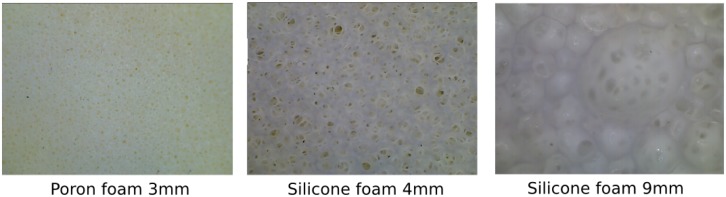
Different foam types and their inhomogeneous structure (the thickness of the material is given in millimeters).

Another parameter is the structure of the foam itself (see Figure 11). The cell structure is not necessarily homogeneous and, thus, can result in different scattering behavior, depending on the position of an optical fiber under the foam.

The described effects demand for a calibration of the sensor span and the sensor offset for each sensor element. Assuming a linear response to incoming forces, the scaling factor can be calculated using the signal span between maximum activation and under no load conditions.

Additionally, the signal range can be used to identify defect sensor elements, as their signal span is much lower than the average signal span of the sensor elements.

### 5.3. Learning Spatial Relationship

In order to reduce the integration effort of the tactile sensor array, the optical fibers are mounted onto the camera chip in a completely disordered way To perceive geometrical object properties of touched objects, it is therefore necessary to find out the spatial relationship of the sensor elements to each other.

The straightforward approach to solve this is to use an activation device that is able to activate a single sensor element. With this device, every sensor element of the field is activated in a predefined sequence and frequency. The activated sensor elements on the camera chips can then be mapped to the sensor elements on the field. The size of such a activation device is dependent on the size of the sensor elements and the spatial density. The higher the resolution, the smaller the activation needs to be in order to avoid cross-activation. Furthermore, the activation needs to be mapped precisely to the arrangement of the sensor field. This requires xyz positioning units, which, again, will not be effective when calibrating sensor shapes on a three-dimensional surface.

A more robust approach that does not rely on any specific calibration tool is exploiting the cross-activation characteristics of the sensor elements. Assuming a single touch activation of the sensor field, all sensors that are activated are assumed to be situated in the same area of the array. Using this property together with the knowledge that no sensor array element can have more than eight neighbors when aligned in a rectangular grid, enables the computation of all neighbors of a sensor element.

The proposed computation can be realized by randomly pressing with a finger on the sensor field. For each sensor element, the activated neighbors are determined together with their signal strength. The way that neighbors of a sensor element are stored follows the way cache memory devices manage their content. Each occurrence of sensor activation is counted and stored. If new sensors are determined as potential neighbors, these are exchanged by the ones with the lowest occurrence. This approach allows the usage of calibration devices that are bigger in size than the distance between neighboring sensor elements. The random activation leads to overlapping activation areas, which, in the long run, will converge to the direct neighbors of each sensor. A good metrics for determining the progress of finding the correct neighbors is the rate at which potential neighboring sensor elements are changed. The slower it is, the more stable the sensor relationships are.

**Figure 12.**
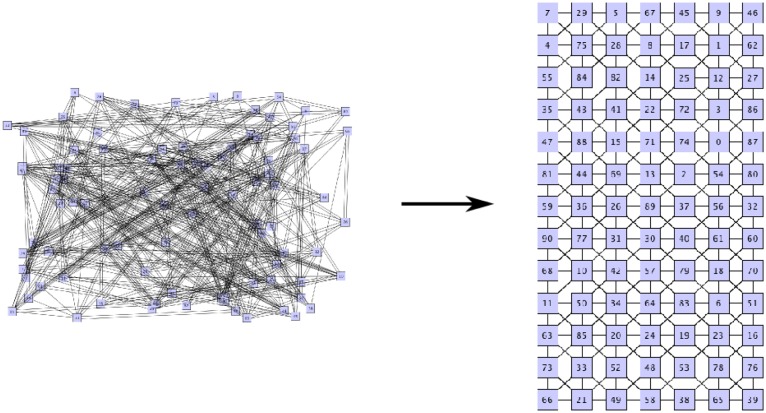
Mapping to the grid of the sensor elements after neighborship detection.

After this algorithm has reached a stable state and the neighbors of each sensor element have been identified, the remaining question is how these sensor elements are arranged with respect to each other. Figure 12 visualizes the open computation step. Although the relationships between the sensor elements are known, the exact location to each other is still unknown.

**Figure 13.**
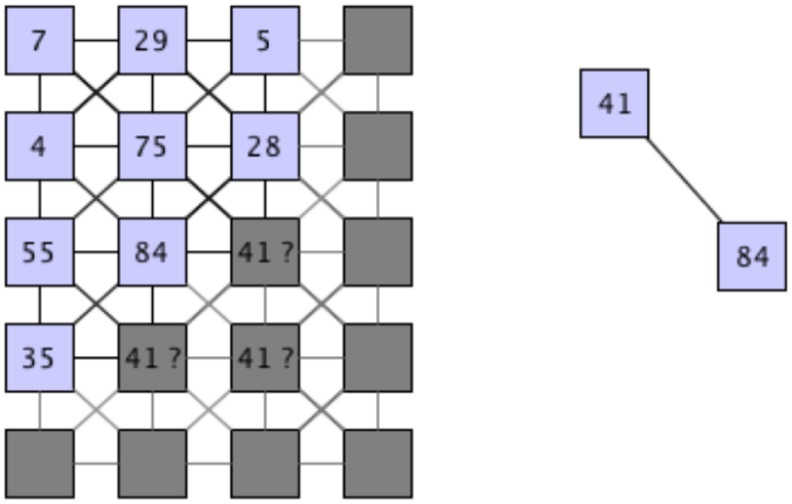
Placement of the neighboring sensor element.

The necessary step is shown in more detail in Figure 13. The sensor element with ID 84 has a known neighbor with ID 41. The question is: where has this sensor element need to be mapped on the grid of the sensor field? This can be solved by comparing the amount of activations on all neighboring nodes, but this can result in high computational effort.

Mapping this question to the field of VLSIplacement techniques during the synthesis of the logic blocks on a chip, like an FPGA, the conditions are quite similar. Logic elements that are connected via wires need to be arranged in such a way that the signal path keeps the timing constraints for the design. Placing components together that are connected with each other is therefore a good approach. A technique that is used to solve this problem is the use of force-directed layout algorithms [20].

The working principle of these algorithms is explained using the sensor element placement challenge described above: Each sensor node can generate attractive or repulsive forces. Sensor nodes that are linked to each other via an edge exert attractive forces and, thus, move towards each other. Sensor nodes exerting repulsive forces are moving away from each other, as they are not interconnected.

**Figure 14.**
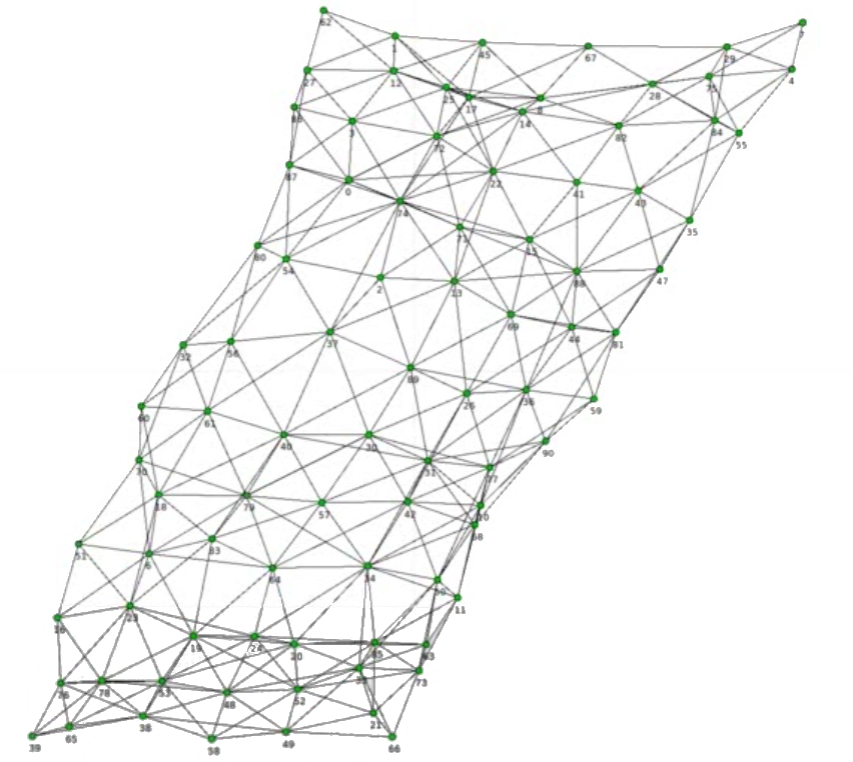
Result of the force-based layout for a sensor field with 91 sensor elements.

This algorithm can be used for aligning the sensor elements in a way that the sensor nodes are mapped to the grid of the sensor field. Figure 14 shows the result of the implementation of the Fruchterman-Reingold force-directed layout algorithm [21] acting on a sensor field after 450 iterations. The algorithm has been applied on a sensor field with 91 sensor elements after 1,440 activation and 605 detected neighborhoods. Three sensor elements were previously detected as defects.

Further improvement on the alignment can be achieved by a subsequent graph matching algorithm that adapts the achieved layout to the final grid alignment known from the manufacturing of the sensor field.

## 6. Sensor Characterization

To benchmark the sensor with respect to properties, like repeatability, creep and sensitivity, a defined measurement environment is necessary to carry out experiments that are repeatable in their setup configuration.

**Figure 15.**
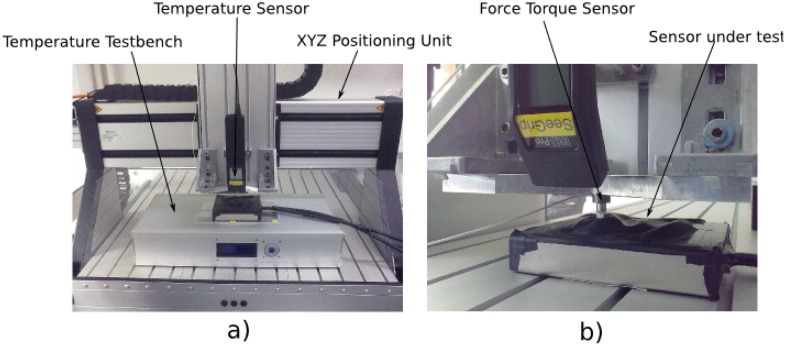
Overview of the developed testbench for tactile sensors (**a**). A detailed view of the probe area (**b**).

A sensor testbench for tactile sensor elements has been developed for this purpose; it is depicted in Figure 15. For measuring the potential temperature dependability of the sensor response, a temperature testbench has been designed. It consists of four Peltier modules, each of which can be operated in a temperature range between −7°C and 100 °C with an accuracy of 0.2 °C. The actual temperature at the sensor element under test is measured by an infrared thermometer that is equipped with an RS232 interface. The thermometer is attached to an XYZpositioning unit that is used to reach each single element on the sensor array. The repeatability of the positioning unit is +/− 0.1 mm. The z-axis of the positioning unit is equipped with a 1 DOF force-torque sensor with a measurement range of +/− 100 N. Attached to this force-torque sensor is an activation device that can be exchanged in order to examine the sensor response when using different kinds of geometries for activating the sensor. All sensor information from the testbench, as well as the sensor data from the sensor under test can be recorded by an analog-digital I/O measurement system.

### 6.1. Measurements

This measurement setup has been used in order to characterize several properties of the fiber-optic sensor. The test device is a sensor consisting of 91 sensor elements with dimensions of seven by four centimeters. A silicone foam with a thickness of four millimeter has been used. The foam has been covered by a black sheet made of LEXAN 700 having a thickness of 260 *μ*m.

The first experiment described has the aim of evaluating the sensitivity and hysteresis of the sensor system. Due to high noise on the force-torque sensor output, it was deemed preferable to use a position-based approach to measure the sensitivity. As the testbench is able to move very precisely in each degree of freedom, the z-axis has been set to activate the sensor module under test with steps of 0.5 mm. The activation depth has been increased in steps up to 2.5 mm and is then released in the same step size.

**Figure 16.**
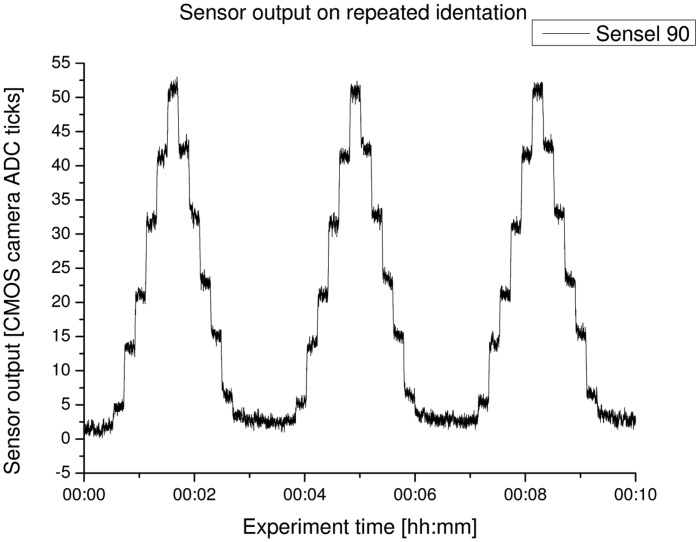
Result of the experiment to test the sensitivity and hysteresis of the sensor.

Figure 16 shows the results of this experiment for a sensor element, which is situated in the center of the sensor module. The different activation steps are clearly visible in the sensor output. Noticeable is the slightly different increase of the sensor signal output, although the activation steps have been of the same size. This can be a hint of the nonlinear behavior of the foam, which acts as the signal conversion between force and light intensity.

Another remarkable observation is that the plateaus of the sensor signal when offloading the sensor are slightly higher than when loading the element. This shows that the foam generates a hysteresis behavior due to the relaxation of the foam material.

Looking at all three iterations of the activation procedure, the measurements indicate a repeatable error of below 0.08%.

**Figure 17.**
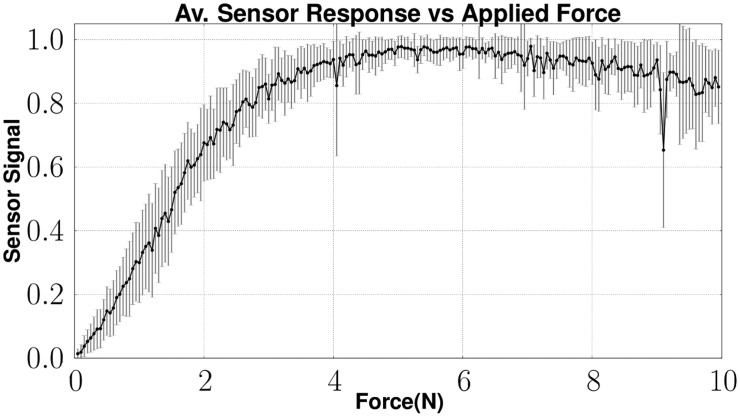
Sensor feedback depending on the applied force.

Figure 17 shows the average feedback and the standard deviation of the same sensor array while increasing the force. The output of the sensor is normalized. While increasing the applied force above 6 N, a decrease in the sensor signal feedback is observable. This effect is caused by the compression of the foam. As the force increases, the foam is squeezed till its complete compression. At this state, the light emitted into the foam structure cannot be sensed by the optical fiber connected to the camera. As this effect slowly increases, the signal feedback decreases.

Figure 8 shows the described effect by looking at the relation between the signal feedback and the compression of the foam. By looking at Figure 17, the sensor signal starts to decrease, while the foam compression is continuing. This is near the maximum compression of the foam.

## 7. Low-Level Processing

The amount of data that is generated in megabytes per second by the complete tactile sensor system is listed in Table 2. As a reference, a gigabit Ethernet connection is able to transmit 119 megabytes per second at maximum. This comparison should motivate that pre-processing sensor data as near to the signal acquisition as possible is favorable.

The described algorithms in the previous sections enable the reduction of the data generated by the acquisition of the fiber optic sensors. Starting from the data generated by six cameras per sensor element, we can reduce the data to the actual sensor element signal aligned to their actual position in the sensor arrays. Together with the data generated by other sensors from different limbs of the gripper or by other modalities, even more processing, analysis and reaction to sensor stimulus can be computed on the gripper hardware.

### 7.1. Experiment

**Figure 18.**
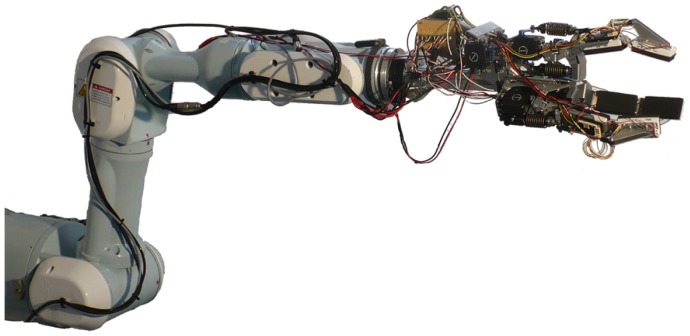
Laboratory setup for experiments on the tactile sensor in-system processing.

How pre-processing of the tactile sensor data can support the implementation of local behaviors has been examined. All experiments haven been carried out using a three-fingered gripper with two limbs each. The morphology of the gripper can be compared to that of a BarrettHand, as the system consists of one fixed finger and two opposable thumbs, each having two limbs. Fixed compliance is realized by having a spring in series with servo motors. The described gripper has been attached to a industry manipulator, like the Mitsubishi PA-10, as shown in Figure 18.

#### 7.1.1. Move Till Contact

This behavior uses the overall output computed by each fiber optic sensor module. The overall output is computed by averaging the output for each sensor. The gripper starts closing until a user defined threshold of the sensor output is reached for every sensor module.

**Figure 19.**
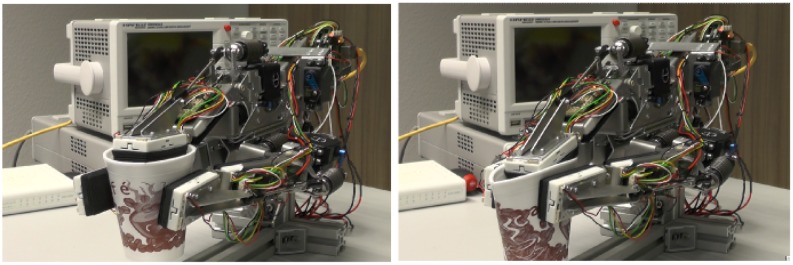
Grasping of a styrofoam mug using in-system processed force-feedback.

Figure 19 shows the result of grasping a styrofoam cup with (left) and without using this feature (right).

**Figure 20.**
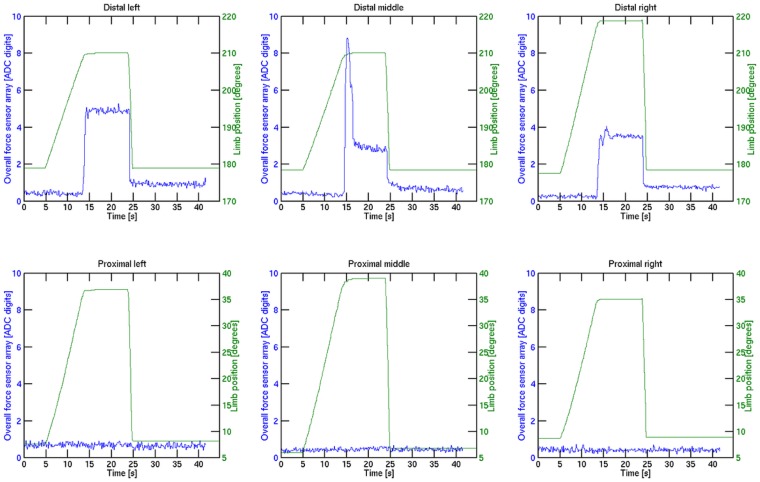
Sensor feedback and joint positions for the behavior that stops movement when having contact at a certain sensor feedback.

The resulting servo and overall output signals for each limb can be seen in Figure 20.

The servo signals shown in green start to increase as the gripper closes the grasp. As the overall sensor output increases its predefined threshold, the servos are locked, and the styrofoam mug remains undamaged in the grasp. After opening the gripper again, the sensor signal decreases. The sensor feedback has a remaining offset. A reason for this could be the restoring forces of the foam and the protective skin. If the alignment of the skin does not allow the complete relaxation of the foam, a constant offset remains, as the foam is still slightly compressed. This fact demands either for a skin that is more flexible or a continuous update of the sensor offsets after each grasp. As other modalities in the system also sense the presence or absence of contact, the latter approach can be realized by reasoning among the other sensor modalities.

## 8. Conclusions

To perceive as much information as possible through the sense of touch, the end-effectors of robotic systems are equipped with sensors that are specialized to measure different modalities.

Besides force-torque sensors and piezoelectric sensors, the presented tactile sensing system for a three-fingered gripper consists of a fiber optic sensor array. The integration of fiber optic sensors into robotic structures can be realized without bulky signal conversion interfaces. The steps, which have been simplified during the mechanical integration, have to be compensated for by more complex software processing using algorithms that can detect and calibrate the sensor elements. All the algorithms presented can be implemented on embedded hardware, like FPGAs. Furthermore, all calibration steps can be performed without any specialized calibration tools, which gives the possibility that those routines can the performed by robotic systems by themselves, given the necessary dexterity.

A simple example of how the low-level processed information can be already used in the end-effector without incorporating higher-level processing systems shows the potential of this approach, which will be extended in the future.

The sensor system proposed here will be crucial for a variety of robots to improve interaction-based control, e.g., of walking robots in terrestrial and space missions ([22,23]). The rich data derived from such sensors can be used to better determine, e.g., the foothold and placement on the ground during the stance phase [24] of walking cycles in legged robots. Such control will, in turn, help to improve the efficiency of walking systems with respect to tracked and wheeled systems.

## Figures and Tables

**Table 1.** Summary of the selected sensors.

**Selected Measurement Principles**				

**Sensing Modality**	**Measurement Principle**	**Chosen Sensor**	**Resolution**	**Update Frequency**
Absolute forces	Strain gauge sensors	ATI nano 25	16-24 bit	100 Hz
Dynamic forces	Piezoelectric sensors	Custom made sensor array	12-24 bit	10 kHz
Force Distribution	Fiber optic sensors	Kinotex sensor transducer	8 bit	30 Hz

**Table 2.** The amount of data generated every second by the proposed sensor system.

**Sensor Type**	**Sampling Rate (Hz)**		**Data Volume (bits)**	**Number of Sensors**	**Output (MB/s)**
Fiber optic sensor	30		640 × 480 × 8	36	316.400
Piezoelectric sensor	10,000		20 × 16	6	2.290
Force-torque sensor	100		6 × 16	3	0.003

Total					318.693
